# Viral Evolved Inhibition Mechanism of the RNA Dependent Protein Kinase PKR's Kinase Domain, a Structural Perspective

**DOI:** 10.1371/journal.pone.0153680

**Published:** 2016-04-18

**Authors:** K. Hari Krishna, Yallamandayya Vadlamudi, Muthuvel Suresh Kumar

**Affiliations:** Centre for Bioinformatics, Pondicherry University, Kalapet, Pondicherry, India; University of Minnesota, UNITED STATES

## Abstract

The protein kinase PKR activated by viral dsRNA, phosphorylates the eIF2α, which inhibit the mechanism of translation initiation. Viral evolved proteins mimicking the eIF2α block its phosphorylation and help in the viral replication. To decipher the molecular basis for the PKR’s substrate and inhibitor interaction mechanisms, we carried the molecular dynamics studies on the catalytic domain of PKR in complex with substrate eIF2α, and inhibitors TAT and K3L. The studies conducted show the altered domain movements of N lobe, which confers open and close state to the substrate-binding cavity. In addition, PKR exhibits variations in the secondary structural transition of the activation loop residues, and inter molecular contacts with the substrate and the inhibitors. Phosphorylation of the P+1 loop at the Thr-451 increases the affinity of the binding proteins exhibiting its role in the phosphorylation events. The implications of structural mechanisms uncovered will help to understand the basis of the evolution of the host-viral and the viral replication mechanisms.

## Introduction

Protein Kinase R (PKR) or Eukaryotic translation initiation factor 2-alpha kinase 2 (EIF2AK2) is an interferon-induced protein, activated by the presence of double stranded RNA (dsRNA) plays a critical role in anti-viral and anti-proliferative defense mechanisms at the cellular levels [1]. PKR belongs to a group of kinases that block the protein synthesis in response to stress signals by the phosphorylation of the α-subunit of translation initiation factor eIF2 [2]. Presence of dsRNA amid viral invasion, cytokine and growth factor deprivation are the principal stress signals induced for the PKR activation [3, 4]. The α-subunit of the translation initiation factor eIF2, a GTP bound protein initiates the first step of the translation mechanism of transferring the methionyl-tRNA to the small ribosomal subunit [5]. PKR blocks protein synthesis by specific phosphorylation of eIF2α at Ser51 modulating the substrate to an inhibitor of its GDP-GTP exchange factor eIF2B [6].

PKR, a 551 amino acid protein consists of a N-terminal dsRNA binding regulatory domain (amino acids 1–170), a C-terminal kinase (amino acids 261–551) catalytic domain and a central region of incognito function. Like all eukaryotic protein kinases, PKR has a smaller, more dynamic amino-terminal lobe (N lobe) and a larger, stable, mostly helical carboxyl-terminal lobe (C lobe) [7]. The N lobe consists of a twisted five-strand antiparallel β sheet (denoted β1 to β5); two helices, α1 and α2 flank the grooves of the β sheet. The C lobe comprises of two paired antiparallel β strands (β7-β8 and β6-β9) and eight α helices (αD to αJ). The activation segment (residues 432–458) positioned between αE and αEF in the lower catalytic lobe serves the Phospho regulatory function. Three very stable helices (αE, αF, and αH) form the core of the C-lobe, whereas the αG-helix, in contrast, is more solvent exposed [8].

Virus, precisely pathogenic forms have evolved novel molecular mechanisms to impair the potent antiviral role of the PKR. Direct interaction with PKR, dsRNA sequestration, PKR pseudo activator and PKR pseudo substrate are the prominent molecular mechanisms of virus involved in countering the PKR role [9]. Proteins K3L of vaccinia virus [10, 11] and TAT of HIV [12, 13] competitively block eIF2α phosphorylation by mimicking the three-dimensional structure of eIF2α and its mode of interaction with PKR.

PKR employs a bipartite mechanism of substrate recognition in recognizing its substrate eIF2α. Phosphorylation sites at Thr-446 and Thr-451 [14] which lie within the activation loop between kinase sub domains VII and VIII play a vital role in the phosphorylation events. T451A mutation inactivates the kinase activity of PKR, while T446A substitution of PKR was partially functional which remains unexplained at the protein structure level [15]. The mechanism by which the viral inhibitors induce the conformational changes and inhibit the PKR interactions remains unclear.

The infections caused by Human Immunodeficiency Virus (HIV) and Hepatitis C Virus (HCV) rank as two of the most important public health problems worldwide [16, 17]. Hundreds of millions of people are infected with either HIV or HCV [18–20], and co-infection with both viruses represents a growing concern that dramatically complicates patient treatment and infection outcome. Understanding the nature and mechanisms of these host pathogen interactions has the potential to unveil novel targets for therapeutic intervention, as well as inform rational vaccine and adjuvant development aimed at protecting against infection by these viruses [21]. We employed Molecular Dynamics Studies (MDS) to understand the molecular interactions and mechanisms involved between PKR and substrate eIF2α including viral inhibitors TAT and K3L.

## Materials and Methods

### Protein Preparation and Docking Studies

The crystal structure of the PKR kinase domain of X-ray structure of the complex of PKR kinase domain-eIF2alpha (PDB entry 2A1A) [22] and the solution structure of the alpha subunit of human eIF2α (PDB entry 1Q8K) [23] were obtained from the PDB database. To model viral PKR inhibitory protein complexes, NMR structures HIV-1 TAT (PDB entry 1TBC) [24] and crystal structure of the K3L protein from Vaccinia virus (PDB entry 1LUZ) [25] were also retrieved from the PDB database. The missing residues of the proteins were filled using the Modeler Version 9.14 software [26, 27] and the resulting structures were minimized for their energy levels.

To investigate the interaction patterns of PKR with substrate eIF2α, inhibitors K3L and TAT we performed, data driven docking with the HADDOCK server [28, 29] which is ranked as best server in both prediction and scoring capabilities at the CAPRI meetings[30]. The interacting residues of the crystal structure 2A1A were used as the active site residues for PKR and the interacting residues of eIF2α Saccharomyces cerevisiae were mapped to Homo sapiens eIF structure using sequence homology. Similarly, the interacting sites of K3L [25] and TAT [31] were retrieved from literature study and docked with the above-mentioned active site of PKR as they inhibit by a competitive inhibition mechanism. The complete lists of the active residues of the proteins involved in the docking were shown in the S1 Table. Passive residues were selected automatically as the exposed surface neighbors of the active residues.

Guru Interface of HADDOCK 2.2 software was used to generate the 1000 models at the first iteration using docking rigid body EM. The best 200 structures (based on energy) were then allowed to perform a second iteration of three simulated annealing refinements. In first iteration, the proteins are considered as rigid bodies and their respective orientation is optimized. In the second simulated annealing, the side chains at the interface are allowed to move. In the third simulated annealing, both side chains and backbone at the interface are allowed to move for some conformational rearrangements. The HADDOCK server in the explicit solvent energy minimization refined the resultant 200 structures. The final stage consists of a gentle refinement by heating the system to 300 K and cooling the system to 100K. The final structures were clustered using the pairwise backbone RMSD at the interface. Upon cluster-structural analysis, 10 lowest energy models were selected, and among these, the best one was characterized based on the lowest HADDOCK score, electrostatic energy and Z-score.

Phosphorylation events of the protein complexes were exercised using the Vienna-PTM 2.0 web server (http://vienna-ptm.univie.ac.at) [32] which has automated the post-translational modifications of the pdb's. Mono-phosphorylation forms (PKR_p_) of PKR complexes were generated by phosphorylating Thr on the 446^th^ residue to phosphothreonine. Double phosphorylation forms (PKR_pp_) were generated by phophorylating Thr on 446^th^ and 451^th^ residues to phosphothreonine similar to the PKR_p_. Six resultant PKR complexes of eIF2α and viral proteins were minimized for the structural variations due to the phosphorylation of the residues were used as the starting point for the MD simulations.

### MD Simulations

MD simulations were performed using the 4.5.5 version of the GROMACS software (www.gromacs.org) [33] using the modified force field gromos54a7 [34] available at Vienna-PTM 2.0 web server (http://vienna-ptm.univie.ac.at). Protein complexes were solvated by SPC (Single Point Charge) [35] water molecules. Adding 4, 11, 2, 6 and 13 Cl^-^ ions respectively neutralized the non-neutral systems of PKR_pp_-K3L, PKR_pp_-TAT, PKR_p_-EIF, PKR_p_-K3L and PKR_p_-TAT. Periodic boundary conditions were implemented in all directions, using a simulation cell with a distance minimum of 1.5 nm from the protein. The non-pair list was updated for every five steps of the md run. The grid neighbor searching method was applied in the simulation with a 1 nm cutoff distance for the short-range neighbor list. Electrostatic interactions were computed using the particle mesh Ewald (PME) [36] summation method with a 1nm short-range electrostatic cutoff. The short-range cutoff used for van der Waals interactions during the simulation was also 1 nm. For the temperature coupling, the solvated structures were divided into two groups (protein and non-protein) using velocity rescaling with a stochastic term. The isotropic pressure coupling was employed in the MD simulation by using the Parrinello–Rahman method [37] with a compressibility of 4.5 × 10^−5^ bar^-1^. During the simulation, constraints were deployed in all bands using the LINC algorithm [38], with parameters LINC-order of 4. First, each system was energy-minimized for 5,000 steps each using steepest-decent and conjugate gradient algorithms; subsequently, the solvent, and ions were equilibrated for 100 ps in constant volume (NVT) and constant pressure (NPT) ensembles, respectively, while the heavy atoms of the protein were restrained harmonically using a force constant of 1000 kJ mol^−1^nm^−2^. Finally, six systems were performed for 50 ns MD simulations after removing all the restraints and all the trajectories were stored every 0.002 ps for further analysis.

### Principal Component Analysis

Principal component analysis (PCA) reveals the functionally important high-amplitude concerted motion of bio-molecules by applying the dimensional reduction method [39]. In MD simulations, the cross-correlation fluctuations between atoms can be described by the covariance matrix. The element C_ij_ in the matrix is defined as:

Cij=〈ΔRi⋅ΔRj〉(〈ΔRi〉2〈ΔRj〉2)12 (1)

where ΔR_i_, ΔR_j_ is the displacement vector corresponding to i^th^, j^th^ atom of the systems and ⟨ ⟩ indicates an ensemble average. PCA diagonalizes the covariance matrix to obtain orthogonal eigenvectors and the corresponding eigenvalues. The eigenvectors, are the principal components (PCs) which represent the directions of the concerted motions. The eigenvalues indicate the magnitude of the motions along the direction. In MD simulations, the first few PCs usually describe the slow and functional motions of a bio-molecular system. In this work, g_covar and the g_anaeig utilities of GROMACS were used to perform PCA to explore and compare the motion of the different complex systems.

### Free-Energy Landscape

The free-energy landscape of a protein obtained using MD simulation technique allows exploring the conformations of proteins in different states. In the FEL, the local basins usually represent the conformational ensemble in the stable states, and barriers indicate the transient states [40–42]. Based on the cosine content, the PCs are chosen as the reaction coordinates to construct the FEL. The relative free energy between two states is calculated by

$$G_{1}\left(X\right)-G_{2}\left(X\right)=-kT\;\text{ln}\left[\frac{P_{1}\left(X\right)}{P_{2}\left(X\right)}\right]$$ (2)

where k is the Boltzmann constant, T is the absolute temperature, the reaction coordinate *X* is the PCs, and *P(X)* is the probability distribution of the system along the PCs. Considering two different PCs *X*_*1*_ and *X*_*2*_ the two-dimensional free-energy landscapes are obtained from the joint probability distributions *P(X*_*1*,_ *X*_*2*_*)* of the system.

### Binding Free Energies of Protein-Protein Complexes

Molecular mechanics Poisson–Boltzmann surface area (MM-PBSA) computational approach was used to estimate binding free energies of protein-protein complexes, which quantifies the strength of a bio-molecular interaction, involved in recognition or catalysis. g_mmpbsa tool inheriting functions from GROMACS as well as Adaptive Poisson-Boltzmann Solver (APBS) was used to calculate energy components [43]. The binding free energy of the protein with protein in solvent can be expressed as

$${\Delta G}_{binding}=G_{complex}-(G_{protein1}+G_{protein2})$$ (3)

where G_complex_ is the total free energy of the protein−ligand complex and G_protein1_ and G_prtoein2_ are total free energies of the isolated proteins in solvent. The free energy for each individual entity can be given

$$G_{x}=\langle E_{MM}\rangle -TS+\langle G_{solvation}\rangle$$ (4)

where x is the protein complex. <E_MM_> is the average molecular mechanics potential energy in a vacuum. TS refer to the entropy contribution to the free energy in a vacuum where T and S denote the temperature and entropy, respectively. The last term <G_solvation_> is the free energy of solvation.

## Results and Discussion

### Protein-Protein Docking and Stability of MD trajectories

The missing residues S351-S354 and S542-A551 of the kinase domain of eIF2α were modeled using Modeler program. The resultant structures of PKR were docked with the eIF2α, K3L and TAT using the HADDOCK software with the mentioned docking procedures. The best structure representing the docked complex was retained based on the lowest HADDOCK score, electrostatic energy, and Z-score. Table 1 shows the HADDOCK scores and energy values of the selected complexes. The docked PKR-eIF2α shows an RMSD of 1.31 Å from the reference pdb structure 2A1A making the dockings of the Haddock server reliable. The selected structures were then subjected to post-translation modification of phosphorylation of T446 and T451 generating the six PKR_p_ and PKR_pp_ complexes. Further analysis was carried on the trajectory files generated from the simulation of the above complexes.

**Table 1:** Statistical analysis of HADDOCK complexes. The statistical values of the selected PKR complexes were indicated along with their cluster numbers. The Z-score indicates the reliability of selected complexes.

Complex	Cluster	HADDOCK score (a.u.)	Cluster size	RMSD from overall lowest energy structure (Å)	Vander Waals energy (E_vdw_)(kcal mol^-1^)	Electrostatic energy (E_elec_) (kcal mol^-1^)	Desolvation energy (E_desol_) (kcal mol^-1^)	Restraints violation energy (kcal mol^-1^)	Buried surface area (Å^2^)	Z-score
PKR-eIF2α	2	-196.1 ±13.6	33	9.9 ± 0.1	-39.9 ± 6.3	-432.3 ± 37.3	41.9 ± 13.1	78.7 ± 32.19	1698.5 ±75.1	-2.1
PKR-K3L	4	-174.6 ±7.8	12	10.0 ± 0.0	-22.4 ± 3.2	-366.3 ± 60.6	39.4 ± 10.0	160.6 ±48.09	1384.5 ±58.9	-1.8
PKR-TAT	1	-199.1 ±5.0	40	8.7 ± 0.1	-38.3 ± 4.8	-248.6 ± 28.7	31.2 ± 13.0	116.2 ±17.35	1562.6 ±225.2	-1.2

**Fig 1:**
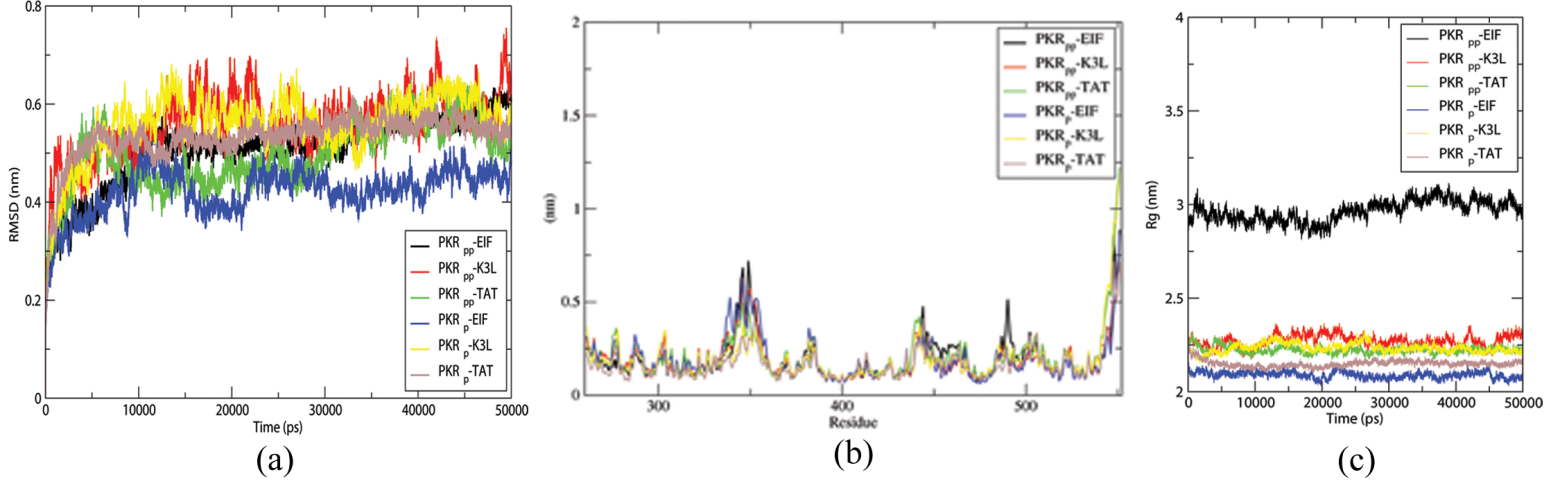
Structral stability of the PKR complexes. The time dependent variations of PKR forms in complex with the substrates and inhibitors. (a) shows the RMSD of the backbone atoms (b) RMSF fluctuation of the PKR residues (c) Radius of gyration of PKR forms in the complexes. Residues showing higher RMSF values are marked in the figure.

The resultant trajectories of the MDS studies were further analyzed using root mean square deviation (RMSD), root mean square fluctuation (RMSF), and radius of gyration (Rg) using g_rmsd, g_sas, and g_gyrate GROMACS utilities respectively, were shown in Fig 1. RMSD and Radius of Gyration (Rg) values of the trajectory files produced a stable plot after 10 ns of simulation with a fluctuation range lesser than 2Å, thus providing the suitability to carry out the further analysis. eIF2α bound PKR_p_ and PKR_pp_ molecules show rmsd plots with lesser fluctuations at 4 Å and 6 Å respectively. The rmsd plots of inhibitor bound complexes show a range of fluctuations ranging from 3 Å to 7 Å except the PKR_p_-TAT form which shows a lesser fluctuation at the 5 Å range due to its smaller surface area. The rmsd plots provide insight how the substrate binding equilibrated the system than the inhibitor molecules.

The RMSF fluctuations calculated on the trajectory of the PKR molecules provide insight to the fluctuations induced by the bound proteins in the PKR molecules. All the complexes show a similar fluctuation pattern independent of the bound protein. The Rg plots of all the protein complexes show a lesser fluctuations ranging from 2.2 Å to 2.3 Å, which shows that the protein complexes are stable. PKR_pp_-eIF2α complex tends to have an Rg value greater than 0.7 nm compared to other complexes showing that the eIF2α binding to PKR_pp_ induces less compactness in the protein complex. Thus, PKR_pp_ maintains short term contacts with the eIF2α substrate in the complex and it can dissociate easily compared to the other mentioned proteins.

### Molecular Interactions of PKR with Substrates and Inhibitors

**Fig 2:**
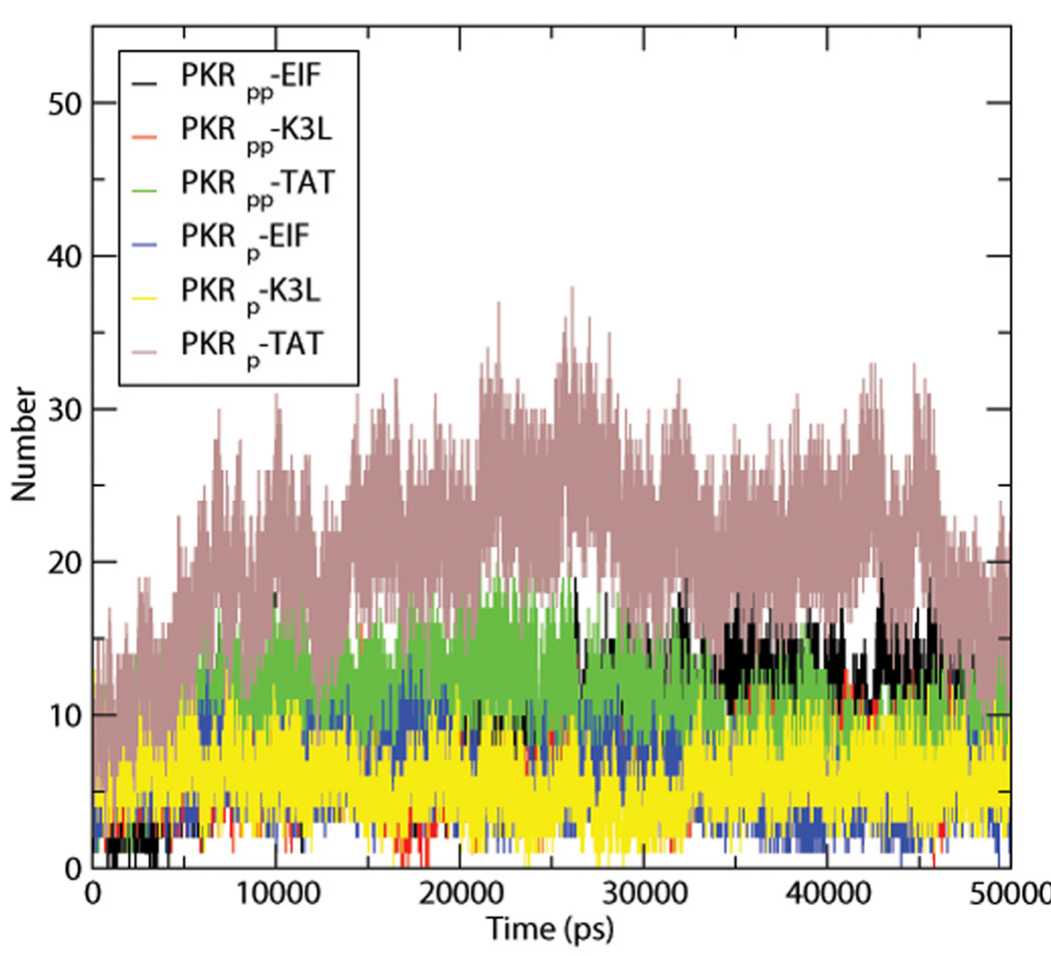
Number of intermolecular hydrogen bonds formed by PKR. The plot showing the number of hydrogen bonds between PKR and the interacting proteins in a time dependent manner.

Hydrogen bonding numbers in protein—protein interactions confers rigidity to the protein structure and specificity of intermolecular interactions. Fig 2 shows the numbers of Hydrogen bonds formed between PKR and proteins in the complex. TAT complex shows an increased number of H-bonds with both forms of PKR. The substrate eIF2α shows a good tendency of H bonding patterns with PKR_pp_ and shows a rapid fall in numbers for PKR_p_. Inhibitor K3L due to the substrate-mimicking feature exhibits an equal number of H-bonds. The TAT protein interacts with the N lobe terminal α-helices and C lobe αD helices showing an increase number of H bond formation than the other protein complexes. The αG lobe and the N lobe terminal α-helices of the PKR_pp_ protein shows an increased H bond formation than the PKR_p_ protein with the eIF2α which contributes to the increased H bonds numbers of PKR_pp_-eIF2α complex. The PKR_pp_-K3L protein complex exhibits H bond formations between phosphorylated Thr 446 and His 47 of the K3L protein. Other protein complexes lack interactions with Thr 446 of the PKR. The double phosphorylated PKR_pp_-eIF2α, PKR_pp_-K3L, PKR_pp_-TAT forms show H-bond formations of phosphorylated Thr with the Arg 54, Arg 56, and Ser57 of eif2α, Lys 45 of K3L; and Lys 28 and Tyr32 of TAT. The phosphorylated Thr 446 and non-phosphorylated Thr 451 PKR_p_ do not show any interactions with the bound substrates or inhibitors at the residual level. Stable hydrogen bonds with greater than ten percent of existence were shown in the S2 Table.

Salt bridges contribute to the protein structure and also to the specificity of protein-protein interactions, were retrieved using the VMD [44] salt bridge plug-in. The plug-in considers the oxygen atoms of acidic residues and the nitrogen atoms of basic residues within the cutoff distance of 3.2 Å in at least one frame for salt bridge formations. The PKR_pp_ forms an increased number of salt bridges with substrate eIF2α than the PKR_p_ involving the N lobe residues Glu-342 and Asp-345,347 residues of the N lobe of PKR. The five stranded β barrel sheet of K3L protein retains the interactions with the C lobe of PKR but fails to interact with the N lobe due to its structural similarity of eIF2α, which reduces the salt bridge numbers in PKR_pp_ and PKR_p_ form. The TAT forms an equal number of salt bridges with the PKR_p_ and PKR_pp_ forms as the substrate eIF2α. The terminal residues of αG helix (residues 497, 499, 500) coordinating with the five-strand β barrel of the interacting proteins shows decreased interactions with the inhibitors K3L and TAT. Glu-28 of eIF2α and Glu-37 of K3L form salt bridges with the activation loop residue Arg-453 of the PKR, in addition Asp-23 of K3L forms salt bridges with the Arg-447 of the PKR’s activation loop. TAT inhibitor lacking the β barrel structure, does not form any charged interactions with the active site residues. Table 2 provides the insight to the salt bridges exhibited by PKR with the interacting protein partners and their percentage of stability.

**Table 2:** Salt bridges formed by PKR with the interacting proteins. Residues of PKR molecules involving in salt bridge formation with the interacting proteins during the course of the simulation. The salt bridges were tabulated along with the numerical values in brackets indicating the distance between N and O atom measured in Å units and percentage of the existence of the salt bridges.

	PKR_p_-eIF2α	PKR_p_-K3L	PKR_p_-TAT	PKR_pp_-eIF2α	PKR_pp_-K3L	PKR_pp_-TAT
N-lobe	ASP 338-ARG 53 (3.3, 2.3)		GLU 269-LYS 71 (2.7, 66.7)	ASP 338-ARG 53 (3.23, 1.5)		GLU 271-LYS 51 (2.9, 2.4)
			GLU 271-LYS 71 (2.81, 22.9)	ASP 338-LYS 60 (2.65, 11.7)		GLU 271-LYS 71 (2.9, 0.3)
			ASP 333-LYS 29 (2.74, 3.8)	GLU 342-ARG 56 (3.29, 3.1)		GLU 335-LYS 28 (2.82, 1.3)
			GLU 335-LYS 29 (2.78, 7.7)	GLU 342-LYS 60 (2.73, 10.3)		GLU 335-LYS 29 (2.82, 0.5)
			ASP 338-LYS 28 (2.7, 82.2)	ASP 345-ARG 52 (3.24, 9.2)		ASP 338-LYS 19 (2.81, 0.6)
			ASP 338-LYS 29 (2.89, 0.7)	ASP 345-ARG 53 (3.26, 22.6)		ASP 338-LYS 28 (2.77, 11.7)
			ASP 338-LYS 19 (2.99, 21.3)	ASP 347-ARG 53 (3.15, 9.5)		ASP 338-LYS 29 (2.72, 29.3)
			ASP 339-LYS 29 (2.79, 1.6)	ASP 347-LYS 60 (2.76, 1.8)		ASP3 39- LYS 29 (2.76, 4.1)
			GLU 342-LYS 19 (2.65, 83.8)			GLU 342 -LYS 85 (2.87, 1.1)
			GLU 342-LYS 85 (2.69, 45.6)			ASP3 47 -LYS 85 (2.77, 6)
			ASP 345-ARG 78 (3.24, 12)			
			ASP 345-LYS 71 (2.78, 3)			
			ASP 345-LYS 85 (2.91, 0.1)			
			ASP 347-LYS 85 (2.7, 29.1)			
			ASP 347-LYS 19 (2.79, 2.3)			
			GLU 349-LYS 85 (2.85, 0.3)			
			LYS 352-GLU 86 (2.07, 11.7)			
P-lobe	GLU 379-ARG 53 (3.26, 3.3)	GLU 375-LYS 74 (2.74, 0.2)	GLU 375-LYS 51 (2.95, 0.5)	GLU 375-ARG 54 (3.04, 29.1)	GLU 375-LYS 74 (2.73, 77.9)	GLU 375-ARG 49 (3.17, 60.4)
	ARG 453-GLU 28 (2.93, 29.4)	GLU 379-LYS 74 (2.85, 3.7)	GLU 379-LYS 50 (2.71, 37.6)	GLU 379-ARG 54 (3.18, 17.2)	GLU 379-LYS 74 (2.77, 24.2)	GLU 375-LYS 51 (2.66, 3.8)
	ASP 486-LYS 79 (2.78, 22)	ARG 447-ASP 23 (3.03, 0.1)	GLU 379-LYS 51 (2.83, 3.4)	ARG 453-GLU 28 (3.29, 15)	GLU 480-LYS 74 (2.75, 57)	GLU 379-LYS 50 (2.77, 10.2)
	GLU 490-LYS 79 (2.79, 4.6)	GLU 480-LYS 74 (2.66, 62.7)	ASP 486-ARG 52 (3.07, 40.1)	ASP 486-ARG 74 (3.21, 13.7)	ASP 500-LYS 22 (2.84, 1.5)	GLU3 79-LYS 51 (2.72, 7.2)
		LYS 493-GLU 37 (2.84, 18.7)	ASP 486-ARG 55 (3.13, 15.1)	ASP 486-LYS 79 (2.83, 6.9)		ASP 486-LYS 50 (2.7, 48.2)
		ASP 500-LYS 22 (2.87, 0.64)	ASP 486-LYS 50 (2.66, 36.4)	GLU 490-LYS 79 (2.68, 9.2)		ASP 486-ARG 52 (3.22, 25.5)
				LYS 493-GLU 42 (2.75, 13.13)		ASP 486-ARG 53 (3.33, 9.3)
				LYS 493-GLU 78 (2.97, 0.03)		LYS 493-ASP 5 (2.83, 1.9)
				ARG 499-GLU 28 (3.05, 4.9)		

Aromatic interactions are important in the structural stabilization of proteins, in protein-protein recognition, and in ligand binding and protein folding. Pdb structure captured at every 5 ns of the simulation was used to predict the aromatic interactions using the PIC web server [45]. The six PKR complexes exhibit the following aromatic-aromatic and cation-pi interactions shown in Table 3. The residue Phe 489 of PKR plays a key role in making aromatic-aromatic interaction with the substrate eIF2α and the inhibitors K3L and TAT. The cation-pi interactions of the PKR-eIF2α complexes were dominated by the interactions with helix αG of the C lobe whereas the inhibitors exhibit a direct interaction with the activation loop residues. The double phosphorylation of PKR reduces the number of aromatic interactions with both the inhibitors but the substrate eIF2α exhibits increased number of contacts due to the conformational changes induced by the phosphorylation at T451.

**Table 3:** Aromatic interactions and Cation-pi interactions of PKR with the interacting proteins. Aromatic interactions between the PKR and the interacting proteins formed during the course of simulation within 7 A°. The cation-pi interactions of PKR with the proteins formed during the course of simulation within 6 A°. The minimum distance between the residues is indicated in the brackets.

Types of interactions	PKR_p_-eIF2α	PKR_p_-K3L	PKR_p_-TAT	PKR_pp_-eIF2α	PKR_pp_-K3L	PKR_pp_-TAT
**Aromatic interactions**						
	Tyr 32-Phe 489 (4.67 Å)	Phe 4-Phe 489 (3.57 Å)	Tyr 47-Phe 489 (5.19 Å)	Tyr 32-Phe 489 (4.36 Å)	Tyr 6-Phe 489 (3.51 Å)	Tyr 47-Phe 489 (4.48 Å)
	Tyr 81-Phe 489 (3.33 Å)	Tyr 6-Phe 489 (3.45 Å)	-	Tyr 81-Phe 489 (3.35 Å)	-	-
	Phe 489-Met 44 (3.34 Å)	Phe 36-Phe 489 (3.9 Å)	-	Phe 495-Met 29 (3.56 Å)	**-**	-
**Cation-pi interactions**						
	Tyr 32-Lys 493 (3.34 Å)	Tyr 24-Arg 447 (3.26 Å)	Tyr 47-Arg 453 (3.26 Å)	Tyr 32-Lys 493 (3.35 Å)	Tyr 24-Arg 447 (3.1 Å)	-
	Tyr 81-Lys 493 (3.26 Å)	Tyr 24-Lys 449 (3.56 Å)	Tyr 346-Arg 78 (3.24 Å)	Tyr 81-Lys 493 (3.57 Å)	Tyr 76-Arg 453 (3.73 Å)	-
	-	Tyr 72-Arg 382 (3.20 Å)	Phe 489-Lys 41 (3.38 Å)	Phe 278-Arg 54 (3.36 Å)	Tyr 454-Lys 74 (4.02 Å)	-
	-	Tyr 76-Arg 453 (4.09 Å)	-	Tyr 346-Arg 52 (3.35 Å)	-	-
	-	Tyr 454-Lys 74 (3.92 Å)	-	Phe 489-Arg 74 (3.23 Å)	-	-
	**-**	**-**	**-**	Phe 489-Lys 79 (2.79 Å)	**-**	**-**

### Binding Energy

In order to illustrate the competitive inhibition mechanism at the molecular level the trajectory files were analyzed for the binding free energies using g_mmpbsa for a time step of 250 ps, thus selecting 200 frames from the trajectory files. The resultant binding energies of six complexes were summarized in the chart. To further analyze the contribution of the PKR molecules to the binding affinity the residual contribution of each PKR residue was summarized in the plots. Both the substrates and inhibitors exhibit higher binding energy with PKR_pp_ forms than the PKR_p_ in their complexes. The TAT form shows an increased binding energy than eIF2α and K3L in both the PKR_p_ and PKR_pp_ forms proving its high inhibition activity. K3L due to its similar structural interaction mechanism of eIF2α shows similar binding energy to PKR_p_-EIF2Α complex.

**Fig 3:**
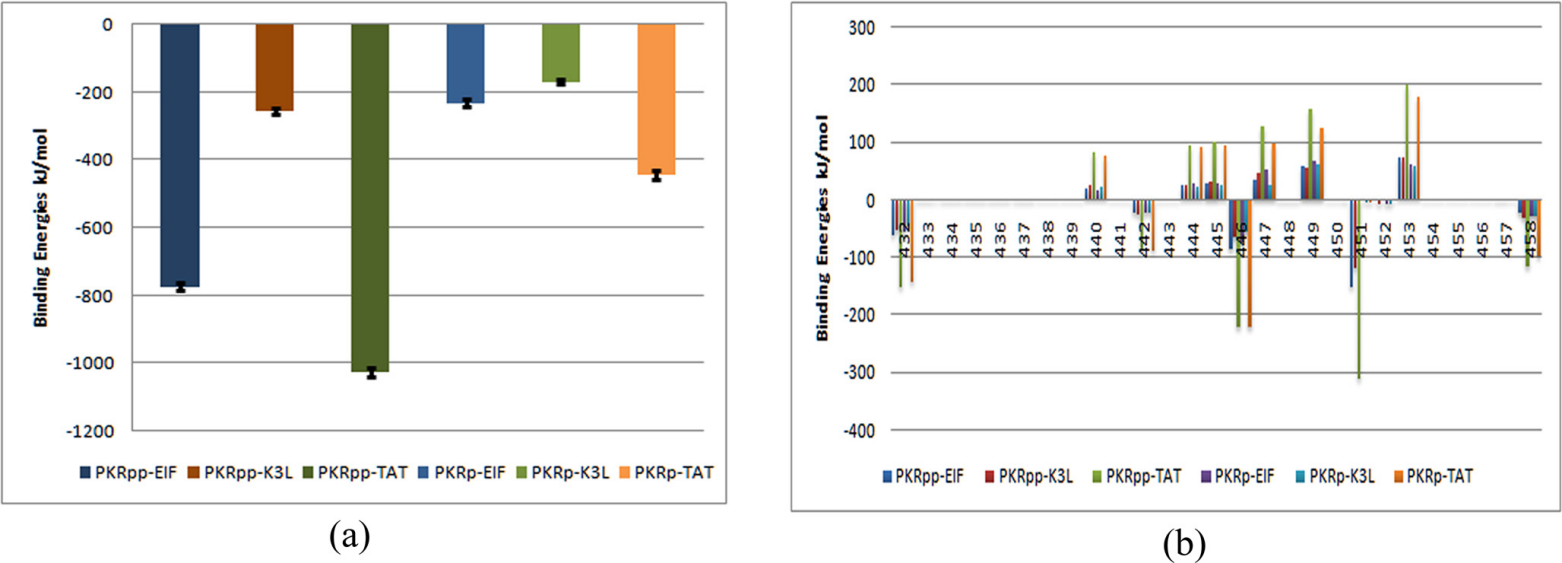
Binding energies calulations of PKR with the substrate and the inhibitors. (a) PKR binding energies with the substrate and the inhibitors. The error bars are indicated by black lines. (b) Residue based decomposition of the binding energies for the activation segment of PKR in the PKR complexes.

To further analyze the role of phosphorylation events of PKR molecules we summarized the contribution to the binding affinity of activation loop residues of PKR to the free energy of binding in the Fig 3. Activation loop residues 432, 442, 446, 451 and 458 show a major contribution to the free energy of binding for the PKR and interacting proteins. TAT shows an increase in the binding energies for the major residues 432, 442, 446 and 458 in both the PKR_p_ and PKR_pp_ forms. Thr 451 phosphorylation shows an increased binding free energy contribution than the non-phosphorylation residues with the same protein complexes. Phosphorylation of Thr 451 residue increases the binding energy of both the substrates and the inhibitors with PKR_pp_ contributing to increased binding energy than the PKR_p_ proteins.

### Secondary Structural Variations in Active Loop

**Fig 4:**
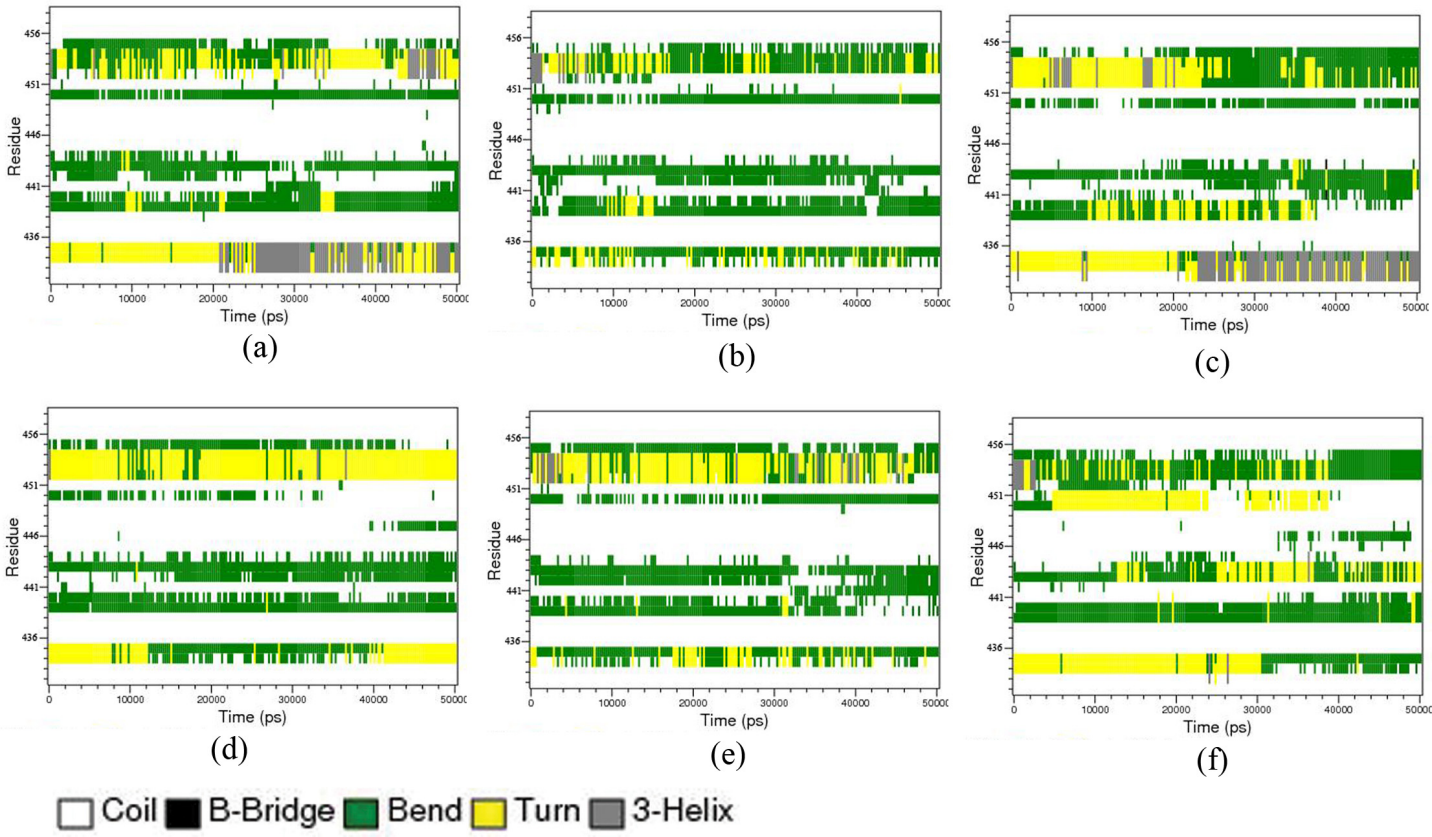
Secondary structural variations of the aloop. The secondary structral variation of the PKR’s aloop ranging from 438–458 residues. The plots are indicated by (a) PKR_pp_-eIF2α, (b) PKR_pp_-K3L, (c) PKR_pp_-TAT, (d) PKR_p_-eIF2α, (e) PKR_p_-K3L and (f) PKR_p_-TAT. The legend indicates the colors used to represent the secondary structural variations.

The capacity of the activation loop to undergo large conformational changes aids the kinase protein to switch between inactive and active states. The activation loop of the PKR protein is highly unorganized with most of the residue residing in the coiled regions. The activation loop harbors the DFG motif, which interacts with the ATP and Mg ions, and P, P+1 phosphorylation sites which play a crucial role in the substrate phosphorylation mechanisms. The major secondary structural transitions visualized using DSSP [46, 47] accounted for the bend to turn transitions and coil to bend transitions to a lesser extent were shown in Fig 4. The DFG motif region of PKR_pp_ bounded to eIF2α and TAT proteins shows a transition from turns to the 3_10_ helices. Other complexes show structural transitions from turns to bends in the DFG motif region, which becomes less compact than the 3_10_ helices. The S3 Table shows the percentage of the secondary structures of both the activation loop and the PKR protein in the complexes. The six protein complexes retain the percentages of the secondary structures irrespective of the PKR’s phosphorylation or the interacting protein in the complexes. The PKR_pp_-eIF2α and PKR_pp_-TAT proteins show an increased percentage of the residues residing in the 3_10_ helices showing the secondary structural alteration of this loop in the above complexes. The P+1 loop residues ranging from 452 to 455 shows structural variations from bend to turn except in PKR_p_-eIF2α complexes where turns were retained. These structural transitions at the P+1 loop help in directing the Thr-451 as a part of the substrate binding groove, directly involved in substrate phosphorylation mechanisms.

The contact surface area between the PKR protein A loop and the binding proteins were calculated using g_sas. The resulting plot shows a steep increase in the residual surface area of the PKR_pp_ interacting with eIF2α from 20 ns, which shows matches with the secondary structural alterations of DFG site. These alterations of DFG site further induce the formation of a 3_10_ helical conformation in the LRY (452–454) residues, which contributes to the increased contact surface area S1(A) Fig. The PDB files of PKR_pp_-eIF2α were clustered based on the RMSD variations of the activation loop with the g_cluster program of GROMACS with a RMSD cutoff of 0.5 nm and using gromos method for cluster determination. These transformations induce Leu 452, Arg 453 and Tyr 454 residues of PKR_pp_ to form novel contacts with the Leu 28 and Arg 29 which are lacking at the start of the simulation as shown in S1(B) Fig. The other complexes retain the coil/bend configuration proving the necessity eIF2α binding and Thr 451 phosphorylation in inducing the secondary structural transitions in the activation loop. These DFG and substrate induced conformational changes of the activation loop promotes the phosphorylation of the substrates by making the P loop and P+1 loop phosphorylation sites accessible to the substrates [48–50].

### PCA of the Protein Complexes

**Fig 5:**
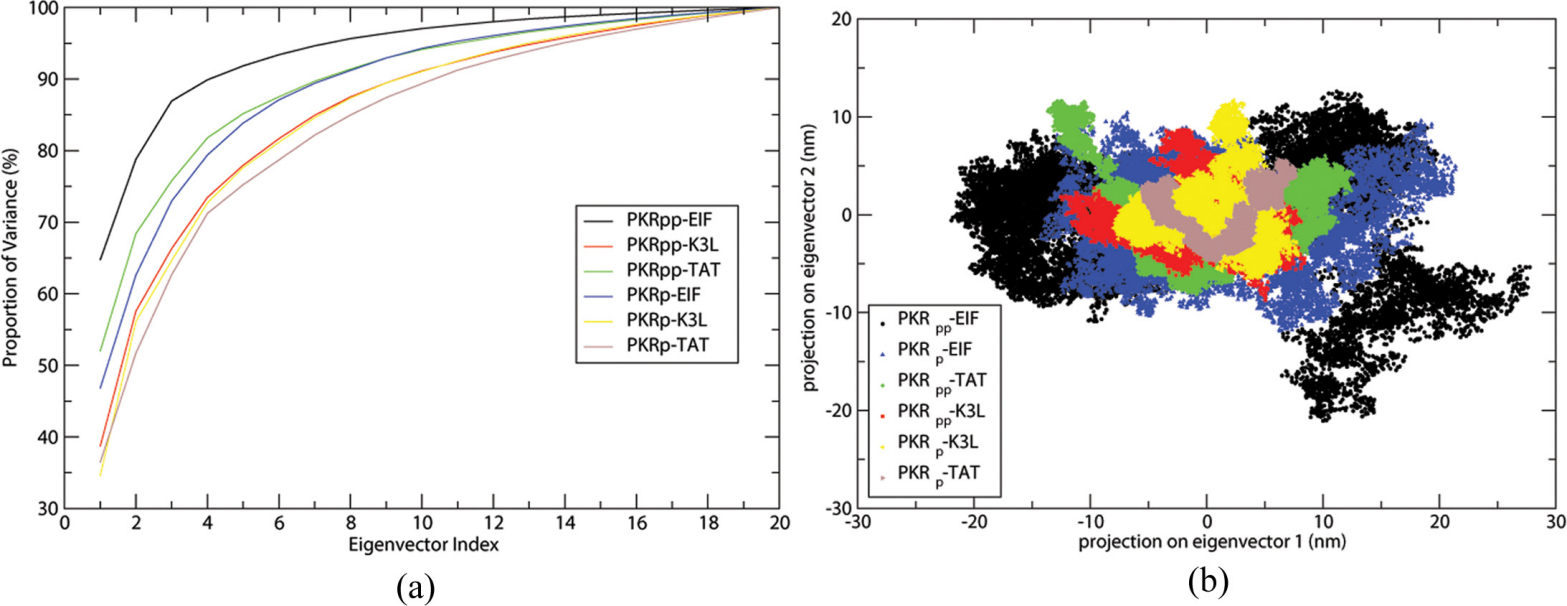
Eigenvector calulations to predict the stability and structral variations of the simulated complexes. (a) Figure showing the cumulative percentages of the proportional variance contributed by the eigenvectors of the PKR complexes. (b) 2D plot of the projections of the eigenvector 1 and eigenvector 2 showing the essential subspace of the complexes.

PCA performed on the simulated ensembles of the six systems as described in methods determines the concerted conformational motions relevant to protein function. The first few eigenvectors from the PCA of the trajectories captured the bulk of the protein dynamics. Fig 5(A) shows the cumulative percentages of the proportional variance contributed by the eigenvectors of the PKR complexes. The first three eigenvectors PKR_pp_-eIF2α complex capture ninety percent of variance in the protein motions. The other protein complexes require greater number of eigenvectors to capture the similar variance in the PKR motions. This shows that the binding of the substrate eIF2α binding with the PKR_pp_ is more stable than the PKR_p_ form. The inhibitors binding with the PKR forms show a relatively lesser variation than the substrate binding, suggesting that the inhibitors form a stable complex with the PKR proteins.

The 2D PC graphs of eigenvector 1 and eigenvector 2 characterizes the essential subspace of the protein complexes, a characteristic of the different folded conformation of the protein [51, 52] were shown in Fig 5(B). The PKR_p_ in complex with the substrate and inhibitor exhibited lower values than the PKR_pp_ complex suggesting that the phosphorylation of the Thr 451 residue has a destabilizing effect on the protein complexes. TAT inhibitor bound to PKR_p_ shows minimal conformation variations due to the few conformational changes it induces in the PKR molecule. The TAT and K3L inhibitors bound PKR occupy a fewer essential subspace in the 2D PC plots than the substrate eIF2α with both the PKR_p_ and PKR_pp_ proteins demonstrating that the inhibitors form a stable complex with the protein PKR irrespective of the number of the phosphorylated sites. The eIF2α bound to PKR_pp_ occupies a larger conformational space than the PKR_p_ protein, which exemplified the structural variations induced by the phosphorylation of Thr 451 in the complexes.

### Free Energy Landscape

**Fig 6:**
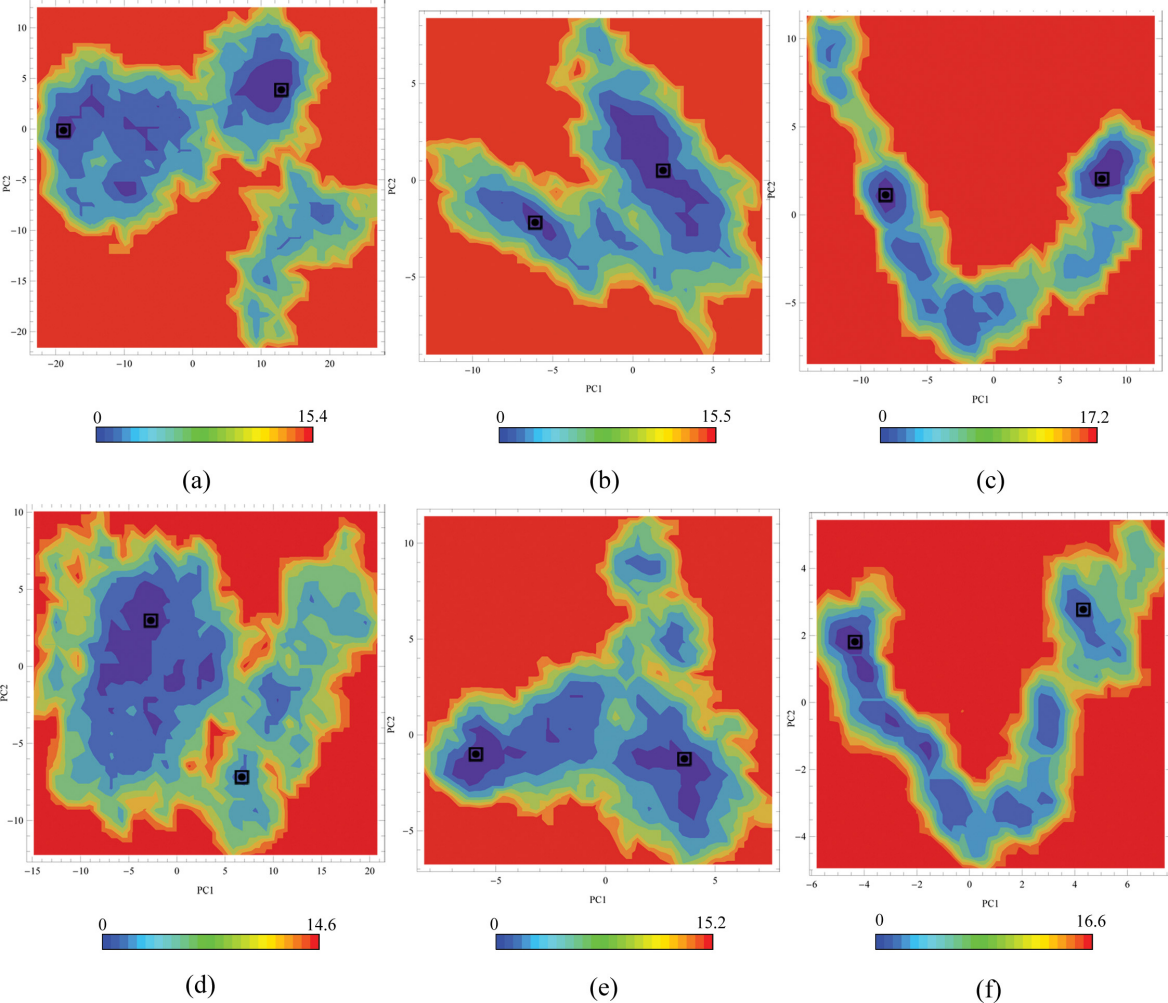
Free energy landscapes of the PKR protein complexes. The plots are indicated by (a) PKR_pp_-eIF2α, (b) PKR_pp_-K3L, (c) PKR_pp_-TAT, (d) PKR_p_- eIF2α, (e) PKR_p_-K3L, (f) PKR_p_-TAT. Conformations having lower energies retrieved from the Free energy landscapes are marked with a dot in the square. The energy bars indicate binding free energy in KJ/mol.

The first two principal component vectors were considered for the construction of the free energy landscapes (FELs). Free energy landscapes of the PKR protein complexes generated were shown in Fig 6. The higher peak in the energy profile reflects the higher stable protein complexes in the simulation trajectory. The FELs were quite different among the six systems; in particular, their energy bins were located in different regions. The PKR_pp_-eIF2α complex shows a two prominent energy bins on the left side and an energy bin with lesser depth energy bins on the right side. The PKR_p_-eIF2α has a widespread energy bin on the left side and a discontinuous energy bin spanning on the right side. The PKR_pp_-K3L has a smaller energy bin on the left side and two-energy bins one spanning lesser area and the other having a wider area with deeper energy bin was on the right side. The PKR_p_-K3L energy bins on the right side have a smaller spanning area and on to the right side was a major spanning bin with discontinuous energy distributions. The TAT complexes with the PKR_pp_ and PKR_p_ have energy bins spanning from the left side to the left with discontinuous energy distributions. The two least energy conformations depicting the major conformational states of the complexes were retrieved from the trajectory file and used for further analysis.

### Electrostatic Interactions of the Binding Surface

**Fig 7:**
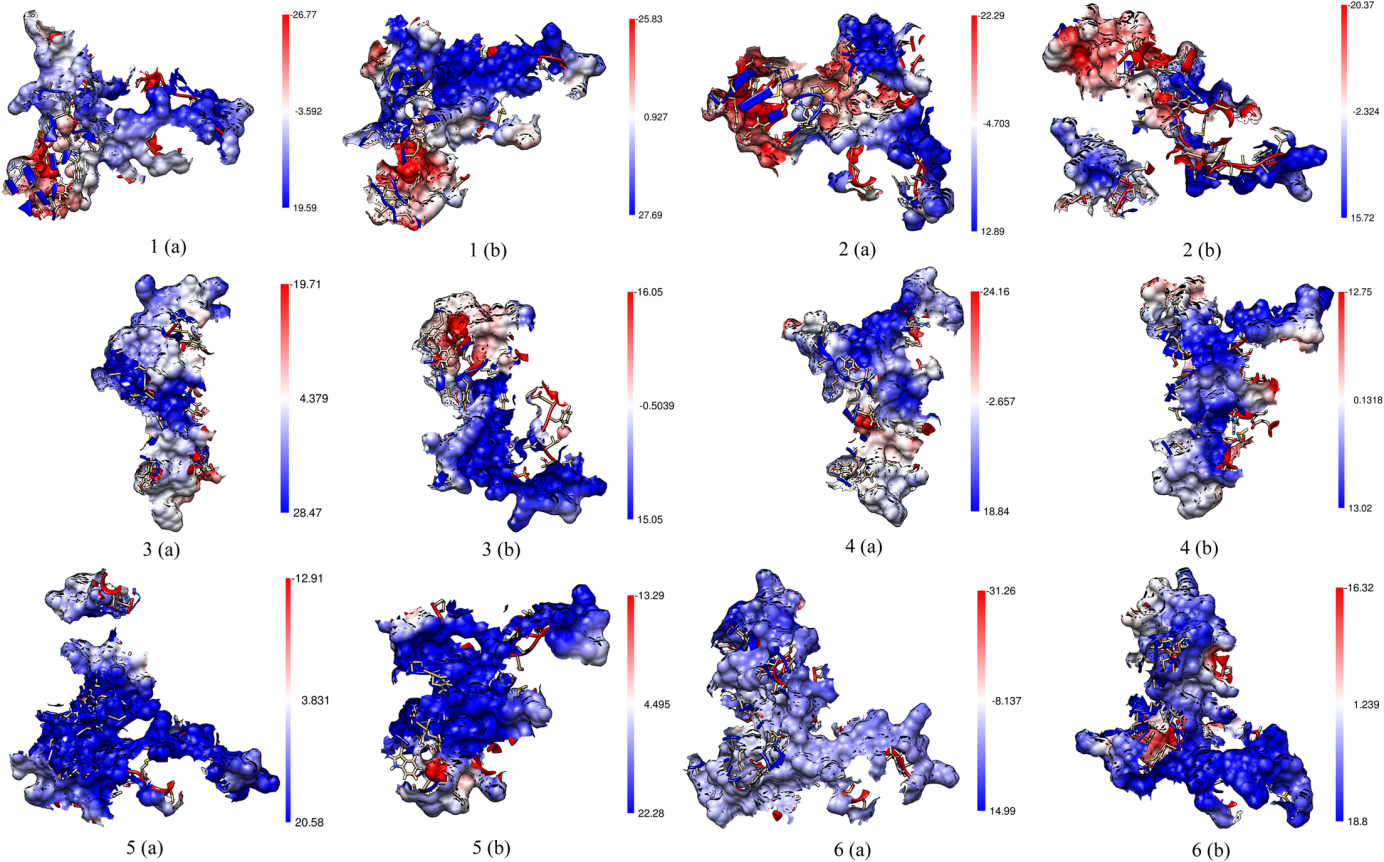
Electrostatic potential maps of the binding surfaces of PKR complexes. The electrostatic potential maps of the binding surfaces along with the activation loop of PKR are shown for the selected stuctres from the free energy landscape plot. Figs 1, 3 and 5 indicate PKRpp bound to eIF2α, K3L and TAT. Figs 2, 4 and 6 indicate PKRp bound to eIF2α, K3L and TAT. The region in red color indicate high electronegative regions and the regions in blue color indicate electropositive regions. The color bars are calibrated in kcal/mol/e.

The electrostatic potential maps of the binding surface area along with the aloop of PKR protein for the above selected structures from free energy landscape plot were generated using the Delphi [53] plugin of UCSF Chimera [54] program. The maps show an altered potentials for the same protein derived from the varied energy bins. The electrostatic potential maps shown in the Fig 7 provide insight to the distribution of the charge potentials on the binding surface. The PKR_pp_ forms show an increased electropositive potential in the PKR interaction surface along with its activation loop than that of the PKR_p_ forms. The eIF2α substrate shows a high-density electronegative region in both the PKR forms, which aids in the retention of the binding interaction with the PKR forms. The inhibitors proteins show a minor electronegativity patch with a lesser variations on the electron density scales. In addition the inhibitors are dominated by a predominant electropositive surface area similar to the PKR forms which inhibits the transfer of a charge moiety. On the other hand, the substrate eIF2α maintains good electro potential balance in both the PKR complexes. The PKR_pp_ form comparable to PKR_p_ form shows an increased electropositive nature due to the presence of additional phosphate group which helps in the phosphorylation of the eIF2α.

### Domain Motion Analysis

**Fig 8:**
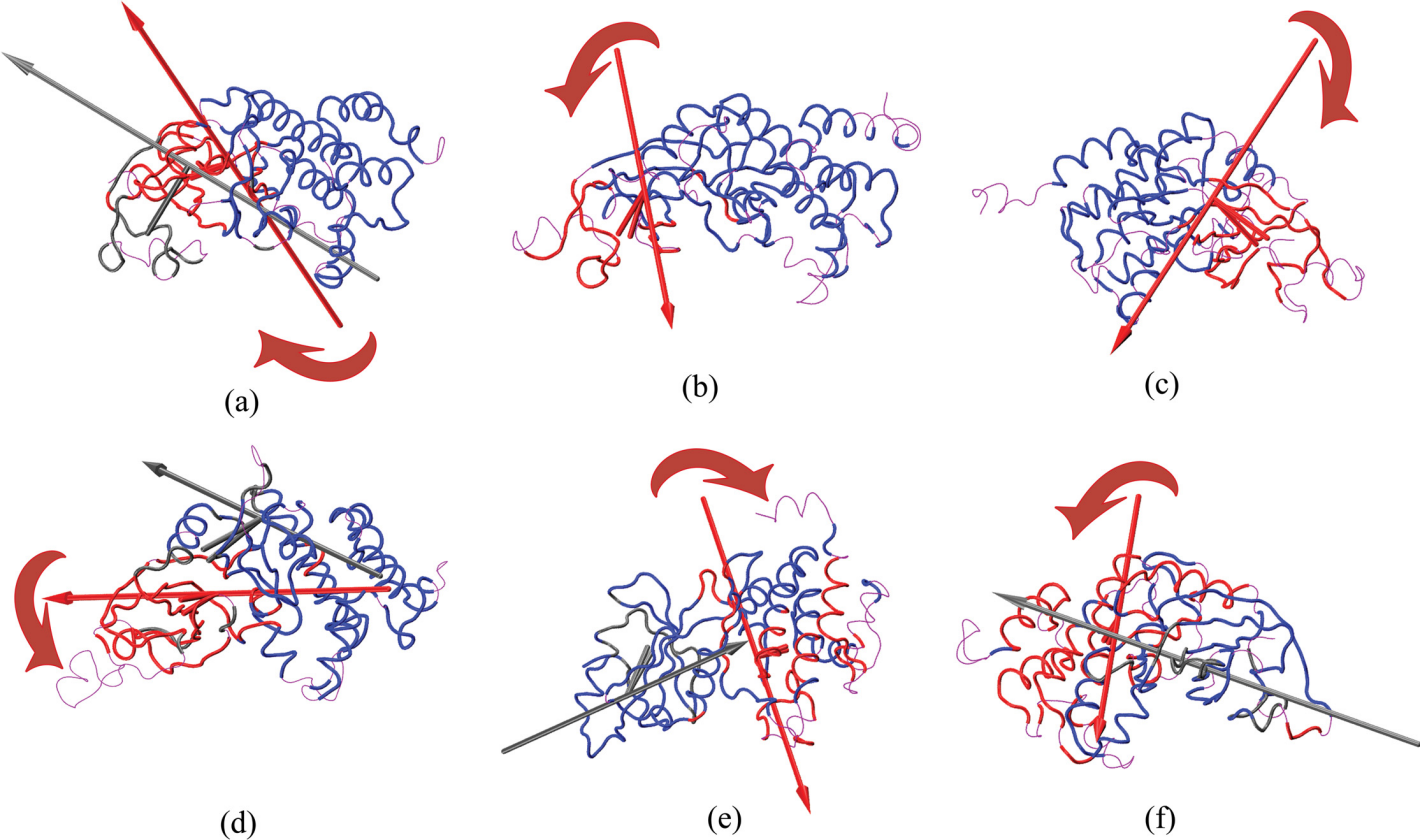
Domain movements of PKR in the protein protein complexes. Effective rotation axes and perpendicular centroid-connecting lines are rendered as tubes in the color of the corresponding domain. The arrows indicate a left-hand rotation, indicating a shift in the center of mass of the domain from the first structure to the second structure. The plots are indicated by (a) PKR_pp_-eIF2α, (b) PKR_pp_-K3L, (c) PKR_pp_-TAT, (d) PKR_p_-eIF2α, (e) PKR_p_-K3L, (f) PKR_p_-TAT. Reference domain, Domain 1 and Domain 2 are indicated by Blue, Red and Black respectively. The arrows drawn indicate the direction of the domain motion.

The substrates and the inhibitors bound to the protein in the complex induce the conformational transitions hinge bending which involves the movement of relatively rigid parts of a protein about flexible joints. Hingefind [55] an algorithm partitions a protein into domains of preserved geometry and subsequently characterizes the relative movements of the found domains by effective rotation axes (hinges). The residues involved in the domain organization are the axis of rotation of the Domain II and I pass through the N lobe of the PKR molecule, which exemplifies that the conformational change of domain opening in the PKR protein was induced, by the flexible N lobes. Fig 8 shows the domain motion of the PKR protein along with their rotational directions.

The four protein complexes PKR_pp_-eIF2α, PKR_p_-eIF2α, PKR_p_-K3L and PKR_p_-TAT organized into Domain 1 and Domain 2, whereas the PKR_pp_ inhibitor complexes with K3L and TAT have a single Domain. The N lobe residues induce a major conformational variation of the PKR in the protein complexes and contribute to a major portion Domain I and Domain II, whereas in PKR_p_-K3L and PKR_p_-TAT the C lobe contributes to the domain organization. The domain motion of PKR in PKR_pp_-eIF2α and PKR_p_-eIF2α complexes were directed away from C lobe with an angular twist of 12.0° and 17.0° respectively inducing an open conformation to the PKR molecule irrespective of the phosphorylation sites. The Domain I of the four-inhibitor complexes of PKR shows an inward movement leading to the closure of PKR binding cavity and inducing a conformational change [56, 57]. The coil regions bridging the β sheets in the N lobe organize to form Domain 2 showing a higher effective angular rotation, which induces the conformational changes in N lobe region. The residues involved in the domain organization and the angular rotation of the domain were mentioned in the S4 Table.

Activation loop of the PKR plays a key role in the molecular phosphorylation events by interacting with the Ser51 of eIF2α. The inhibitors bound to the PKR forms induce an inward closure domain motion, which pushes the activation loop inside, making it inaccessible to the substrates further binding with the PKR forms. The substrate binding induces an open conformation in the PKR form which pushes the activation loop towards the interacting substrate protein, which exposes the activation loop phosphor donor site to the Ser 51 of eIF2α aiding in the phosphorylation. The Thr 551 additional phosphorylation induces an increased conformational variation of 5° than the mono phosphorylation Thr 446 making it a favorable substrate phosphorylation site.

**Fig 9:**
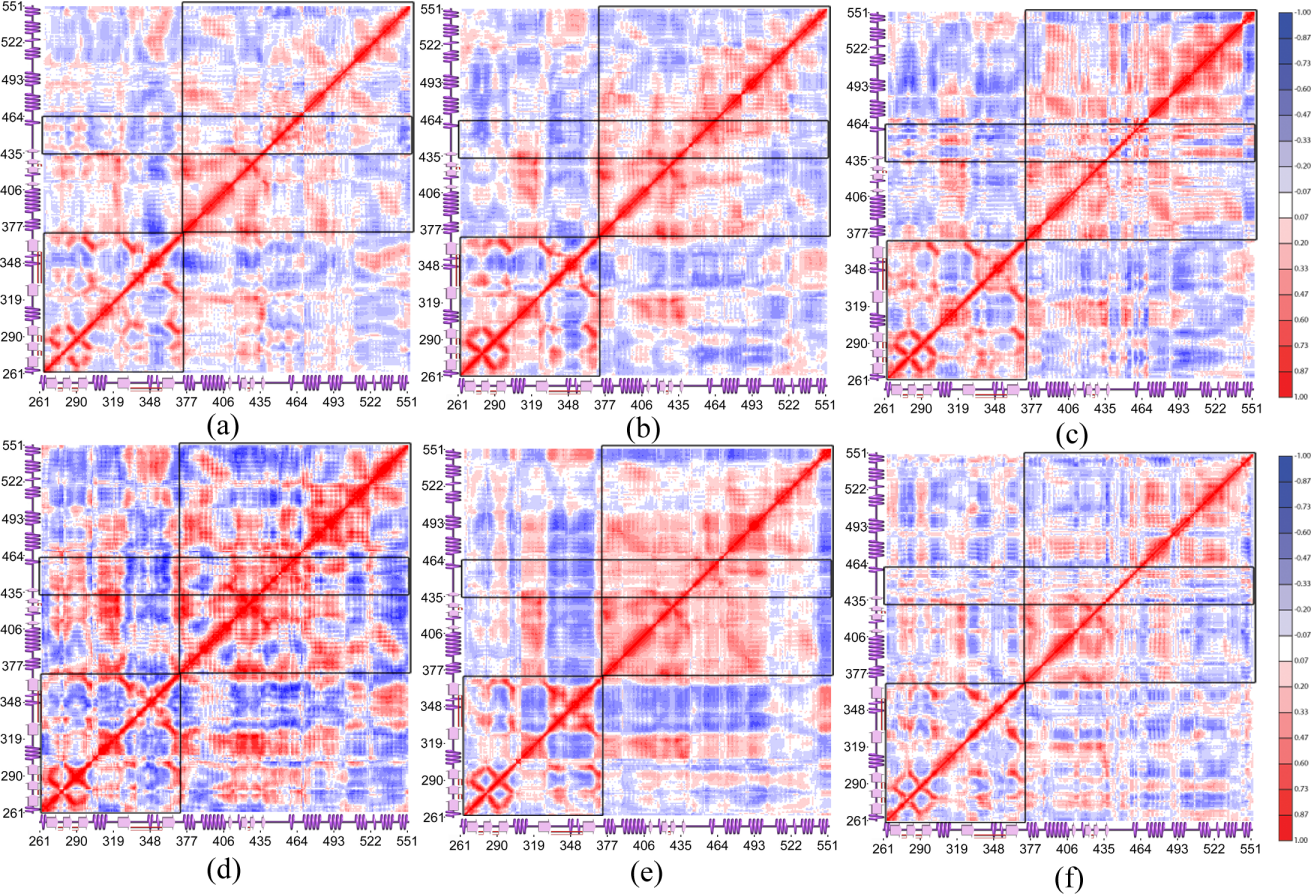
Cross-correlation heat maps of the PKR protein. Cross-correlation heat map generated using the PCA vectors showing the correlated and anti-correlated regions in the protein structure. The plots are indicated by (a) PKR_pp_-eIF2α, (b) PKR_pp_-K3L, (c) PKR_pp_-TAT, (d) PKR_p_-eIF2α, (e) PKR_p_-K3L, (f) PKR_p_-TAT. The P, N lobes and the activation segment are demarked by a boxed structure.

To further analyze the altered domain motion of the PKR protein’s N and C-lobe upon interacting with the substrate or inhibitor, a cross-correlation heat map was generated based on the PCA vectors using ProDy python package [58]. Comparing the cross-correlation matrices of different simulation trajectories reveal the similar features shown by the relative motions. Fig 9 shows the cross-correlation matrices of the six protein complexes generated using the PCA eigenvectors. Many regions of the protein, especially the regions of secondary structure, move in a correlated manner [59, 60]. A higher degree of cross correlation among the residues was observed in the PKR_p_-eIF2α complex followed by the PKR_p_-K3L complex.

The N-lobe of all the protein complexes shows a similar inter-residual correlation whereas the C-lobe shows higher variations in inter-residual correlations due to its interactions with the substrate or the inhibitor protein. The inhibitors’ binding induces a higher degree of negative correlation in the internal domain motions of the PKR’s C-terminal residues; in comparison, the substrate binding shows a higher degree of positive correlation in comparison to the inhibitor bound complexes. A similar induced negative correlation motion was observed in the activation segment residues in the inhibitor complexes with the C-lobe region and a lesser extent with the N-lobe region. The αG which interacts with the protein partners shows a positive correlation with the activation loop residues only in the PKR_pp_-eIF2α complex and all the other protein complexes show the negative correlation.

The correlated domain motions of the PKR protein provide insight to alter domain motions of PKR in relation to the secondary structural features. The substrate binding and the phosphorylation events of the Thr-451 decreases the negative correlation between the secondary structure elements, whereas the inhibitors binding and non-phosphorylation of Thr-451 relatively induces the negatively correlated movements in the secondary structure elements of the PKR. These positively correlated movements of the PKR elements induced by the eIF2α binding play a key role in directing the N-lobe and C-lobe an outward motion aiding in the projection of the activation segment.

## Conclusion

Auto phosphorylation of the PKR is a post-translational modification, which activates the protein through multiple phosphorylations of Ser and Thr residues by autocatalysis. Multiple auto phosphorylation events appear to be an integral part of eIF-2AK family protein kinase function. Contemplating the potential role of the phosphorylation sites in the functional mechanism of the kinases will help in understanding the activation and inhibition mechanisms of eIF2-AK family kinase proteins. Thr 551 residue in addition to Thr 446 residue phosphorylation increase the interactions of PKR with eIF2α in numbers of the H bond, salt bridge and aromatic interaction formed by C-lobe and activation loop of PKR with the five stranded β barrel.

The inhibitor interacted with the PKR molecules by forming stable salt bridges, H-bonds and aromatic interactions comparable to the substrate eIF2α. Five stranded β barrel structure of K3L forms stable interactions with the αG loop of the PKR molecule. TAT protein inhibits in a distinct mechanism of interactions with the PKR spanning from N-lobe to C-lobe. Distortions induced by phosphorylation of Thr-451 in the αG helix of PKR_pp_ forms alters its interaction pattern with the viral inhibitors by decreasing its interactions with the distorted αG helix and the activation loop residues.

The inhibitor interaction with the varied phosphorylation PKR forms shows much stability with a fewer conformational alteration whereas the substrate eIF2α shows a much varied conformational changes in the PCA plots. Though the inhibitors show lesser interactions than the substrate, the inhibitor protein complexes were stabilized with higher binding energies from the PKR protein, which involves large amounts of interaction energy contributed by the non-bonded interactions of the activation segment residues significantly by the phosphorylation of Thr 451 residue.

PC analysis of the PKR complexes gives insight to the higher conformational flexibility induced in the substrate bound complexes than the inhibitors, which prefer a stable configuration with limited conformational changes. The substrates and the inhibitors induce an altered opening and closing domain motion by inducing changes in the C-lobe of PKR protein. The inhibitor characteristic properties of inducing the conformational stability and closing domain motion offer an insight to the competitive inhibition of PKR by viral proteins and its structural alteration. The PKR_pp_ complexes with eIF2α and TAT molecules show a functional transition from turns to 3_10_ helices in the DFG motif region, whereas a similar transition was seen in the P+1 loop which interacts directly with the substrate was viewed in PKR_pp_-eIF2α but absent in other substrates.

The present works emphasizes on the loss or gain of contacts due to the altered phosphorylation of Thr 446 and Thr 451 residues of PKR and predicts domain motions based on the resultant structural variations. To understand the exhaustive phosphorylation and inhibition events by the substrates or the inhibitors an extensive simulation at a millisecond scale and quantum chemical calculations have to be applied on the protein complexes. The events involved in phosphorylation of eIF2α by PKR and its inhibition of cellular translation mechanism remain vague. An insight into the inhibition mechanisms evolved by the virus to control the above process will help to regulate the viral replication mechanisms in host tissues. The molecular dynamics studies provide insight into the role of auto phosphorylation of Thr 451 in substrate and inhibitor recognition mechanisms. Varied interaction patterns of viral inhibitors with PKR_p_ and PKR_pp_ forms discussed will help us to understand the host-viral interaction mechanisms.

## Supporting Information

S1 Fig Contact surface calulations between PKR and the interacting proteins. (a) The time course vaiation ofthe surface area in contact between the PKR and the interacting prtoeins. (b) The newly formaed contacts between the PKR and eIF2α due to the indcution of 3_10_ helix in the DFG loop
(TIF)

S1 Table Active residues of proteins involved in docking. The residues of the proteins tabled below are defined as the active site residues for protein-protein docking in the HADDOCK server.
(DOCX)

S2 Table Hydrogen bonds formed by PKR protein in the six complexes. Hydrogen bonds formed between the PKR protein and interacting proteins having more than ten percentage of existence during the course of the simulation. The values indicate the percentage of existence of the Hydrogen bonds.
(DOCX)

S3 Table Secondary structural variations of PKR protein. The percentages of PKR residues and the Aloop residues in different secondary structural regions in the six protein complexes. A major change can be observed in the 3_10_ helix formations of the Aloop.
(DOCX)

S4 Table Domain motion contributing residues. Residues involved in the domain formation, which helps the PKR protein to rotate, and their effective rotational angles are indicated.
(DOCX)
