# Supplementary figures and images for: Viral Evolved Inhibition Mechanism of the RNA Dependent Protein Kinase PKR's Kinase Domain, a Structural Perspective
Source: PLoS One. 2016 Apr 18;11(4):e0153680. doi: 10.1371/journal.pone.0153680 (PMC4835081; doi:10.1371/journal.pone.0153680)

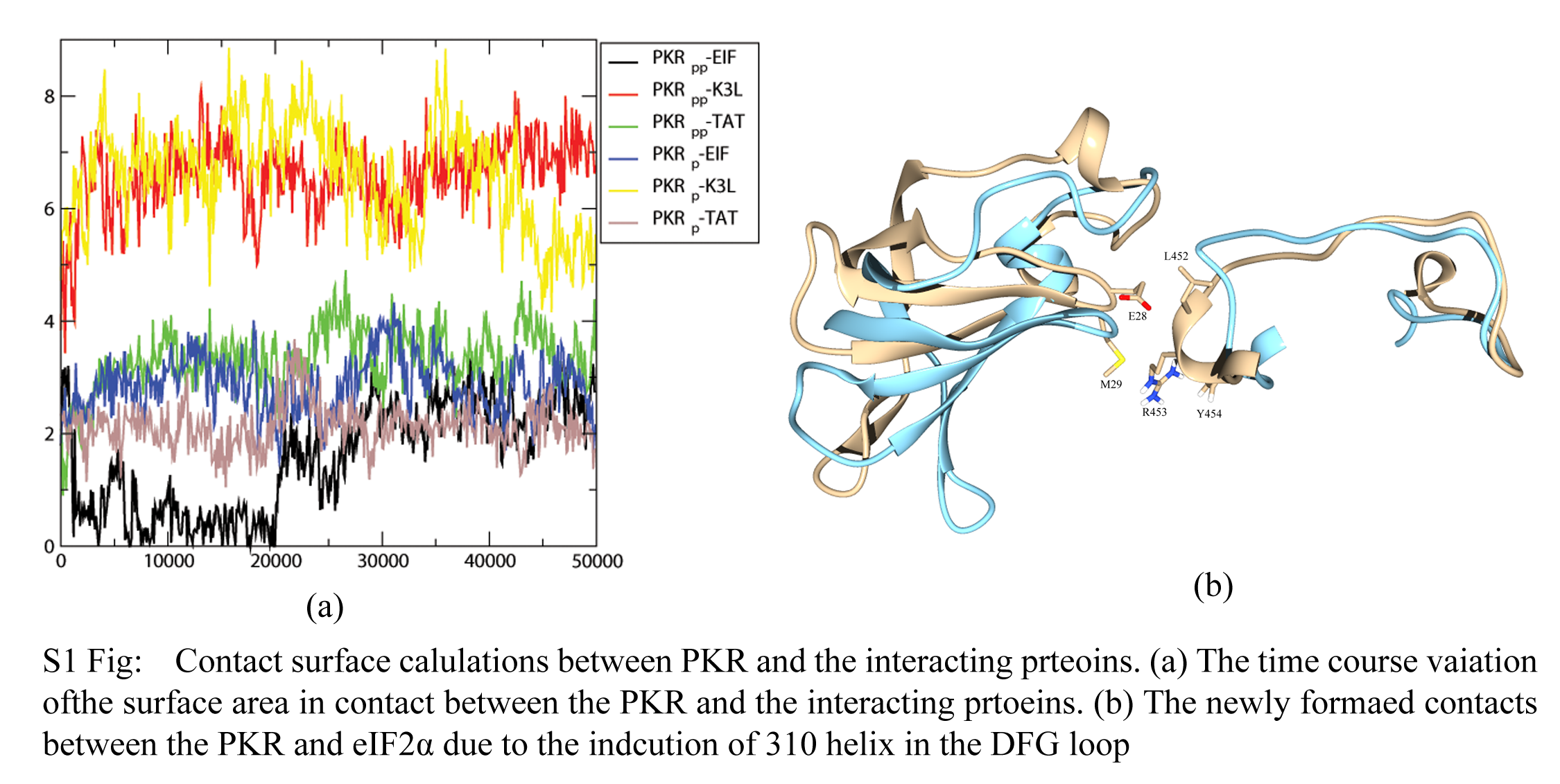

Supplement: S1 Fig — (a) The time course vaiation ofthe surface area in contact between the PKR and the interacting prtoeins. (b) The newly formaed contacts between the PKR and eIF2α due to the indcution of 310 helix in the DFG loop (TIF) [file pone.0153680.s001.tif]
