# Supplementary material for: Viral Evolved Inhibition Mechanism of the RNA Dependent Protein Kinase PKR's Kinase Domain, a Structural Perspective
Source: PLoS One. 2016 Apr 18;11(4):e0153680. doi: 10.1371/journal.pone.0153680 (PMC4835081; doi:10.1371/journal.pone.0153680)
Supplement: S1 Table — The residues of the proteins tabled below are defined as the active site residues for protein-protein docking in the HADDOCK server. (DOCX) [file pone.0153680.s002.docx]

**S1 Table. Active residues of proteins involved in docking**. The residues of the proteins tabled below are defined as the active site residues for protein-protein docking in the HADDOCK server.

| **Protein** | **Active residues** |
| --- | --- |
| PKR | Glu 379, Arg 382, Thr 451, Leu 452, Arg 453, Asp 486, Thr 487, Ala 488, Phe 489, Glu 490, Ser 492, Lys 493 |
| eIF2α | Glu 28, Met 29, Tyr 32, Glu 42, Gly 43, Met 44, His 46, Arg 74, Asp 76, Lys 79, Tyr 81, Ile 82, Asp 83 |
| K3L | Gly 75, Tyr 76, Ile 77, Asp 78 |
| TAT | Thr 39, Leu 43, Ile 45, Ser 46, Lys 51, Arg 52, Arg 53, Arg 55 |
