# Supplementary material for: Viral Evolved Inhibition Mechanism of the RNA Dependent Protein Kinase PKR's Kinase Domain, a Structural Perspective
Source: PLoS One. 2016 Apr 18;11(4):e0153680. doi: 10.1371/journal.pone.0153680 (PMC4835081; doi:10.1371/journal.pone.0153680)
Supplement: S2 Table — Hydrogen bonds formed between the PKR protein and interacting proteins having more than ten percentage of existence during the course of the simulation. The values indicate the percentage of existence of the Hydrogen bonds. (DOCX) [file pone.0153680.s003.docx]

**S2 Table. Hydrogen bonds formed by PKR protein in the six complexes.** Hydrogen bonds formed between the PKR protein and interacting proteins having more than ten percentage of existence during the course of the simulation. The values indicate the percentage of existence of the Hydrogen bonds.

| **PKR_pp_-eIF2α** | **PKR_pp_-K3L** | **PKR_pp_-TAT** | **PKR_p_-eIF2α** | **PKR_p_-K3L** | **PKR_p_-TAT** |
| --- | --- | --- | --- | --- | --- |
| GLU 271 OE2 - ARG 53 NH1 (10.176) | GLU 375 OE2 - THR 73 OG1 ( 23.887 ) | ILE 273 O - ARG 49 NE ( 23.987 ) | GLN 280 NE2 - ARG 54 NH1 ( 10.896 ) | SER 275 OG - LYS 45 NZ ( 10.668 ) | GLU 269 OE1 - LYS 71 NZ ( 14.763 ) |
| SER 337 O - SER 57 OG (35.523) | GLU 375 OE1 - LYS 74 NZ ( 16.055 ) | GLY 274 N - ARG 49 NH2 ( 22.191 ) | TYR 332 OH - ARG 54 NH2 ( 14.963 ) | GLY 277 N - VAL 44 O ( 21.739 ) | GLU 271 OE1 - LYS 71 N ( 40.67 ) |
| GLU 342 OE1 - ARG 56 N (40.594) | GLU 379 OE1 - THR 73 OG1 ( 25.047 ) | GLN 280 NE2 - LYS 29 O ( 41.254 ) | LEU 452 N - GLU 28 OE1 ( 33.011 ) | GLU 303 OE2 - HIS 47 NE2 ( 12.34 ) | GLY 276 N - HIS 33 ND1 ( 43.618 ) |
| GLU 342 OE1 - SER 57 N (44.014) | GLU 379 OE2 - LYS 74 NZ ( 42.438 ) | SER 337 OG - HIS 33 NE2 ( 14.343 ) | ARG 453 NE - GLN 27 NE2 ( 18.147 ) | GLU 335 OE1 - HIS 47 NE2 ( 16.203 ) | GLN 280 OE1 - HIS 33 NE2 ( 31.267 ) |
| GLU 342 OE1 - SER 57 OG (32.387) | T2P 446 OG1 - HIS 47 NE2 ( 14.991 ) | GLU 375 OE1 - ARG 49 NE ( 24.739 ) | ARG 453 N - GLU 28 OE1 ( 55.814 ) | GLU 375 OE1 - LYS 74 NZ ( 13.411 ) | SER 337 OG - LYS 19 NZ ( 14.439 ) |
| SER 343 OG - ARG 53 O (23.379) | T2P 446 OE1 - HIS 47 NE2 ( 10.124 ) | GLU 375 OE1 - ARG 49 NH2 ( 22.051 ) | ARG 453 NE - GLU 28 OE2 ( 45.426 ) | ILE 378 O - THR 73 OG1 ( 12.715 ) | SER 337 OG - ASN 23 ND2 ( 14.723 ) |
| T2P 451 N - ARG 54 NH2 (18.511) | LYS 449 NZ - MET 46 O ( 13.691 ) | GLU 379 OE1 - LYS 51 N ( 25.015 ) | ARG 453 N - GLU 28 OE2 ( 33.651 ) | GLU 379 OE2 - TYR 72 OH ( 23.307 ) | SER 337 O - LYS 28 NZ ( 27.715 ) |
| T2P 451 OE3 - ARG 56 NE (34.319) | GLY 450 O - TYR 76 OH ( 52.486 ) | GLU 379 OE1 - ARG 52 N ( 31.783 ) | ARG 453 NE - GLU 28 OE1 ( 16.615 ) | SER 418 OG - LYS 74 NZ ( 17.563 ) | SER 337 N - LYS 29 O ( 36.371 ) |
| T2P 451 OE2 - ARG 56 NE (10.332) | T2P 451 N - LYS 45 NZ ( 19.399 ) | ARG 382 N - ARG 52 NH1 ( 15.111 ) | ARG 453 NH2 - GLU 28 OE2 ( 12.955 ) | LYS 449 NZ - LYS 45 O ( 16.747 ) | SER 337 OG - CYS 30 O ( 10.192 ) |
| T2P 451 OE3 - SER 57 OG (41.606) | T2P 451 OG1 - LYS 45 NZ ( 15.555 ) | SER 418 OG - LYS 51 NZ ( 20.915 ) | ARG 453 NH1 - GLU 28 OE2 ( 10.056 ) | LEU 452 N - TYR 27 OH ( 13.235 ) | SER 337 OG - TYR 32 O ( 20.171 ) |
| LEU 452 N - GLU 28 OE1 (14.859) | T2P 451 OE1 - LYS 45 NZ ( 12.196 ) | T2P 451 N - LYS 28 NZ ( 27.607 ) | SER 492 N - GLN 27 NE2 ( 69.969 ) | ARG 453 N - TYR 27 OH ( 19.603 ) | ASP 338 OD2 - LYS 19 NZ ( 13.347 ) |
| ARG 453 N - GLU 28 OE1 (21.863) | LEU 452 O - TYR 76 OH ( 16.211 ) | T2P 451 OE1 - LYS 28 NZ ( 17.907 ) | LYS 493 NZ - GLU 42 OE2 ( 12.264 ) | ARG 453 N - TYR 76 OH ( 28.231 ) | ASP 338 OD1 - ASN 23 N ( 53.814 ) |
| ARG 453 N - GLU 28 OE2 (20.023) | LEU 452 N - TYR 76 OH ( 40.41 ) | T2P 451 OG1 - LYS 28 NZ ( 12.671 ) | THR 496 N - GLN 27 OE1 ( 21.127 ) | TYR 454 OH - LYS 74 NZ ( 12.368 ) | ASP 338 OD1 - ASN 23 ND2 ( 28.011 ) |
| ARG 453 NE - GLU 28 O (19.715) | ASP 486 OD1 - CYS 5 N ( 22.183 ) | T2P 451 OE2 - TYR 32 OH ( 39.282 ) |  | GLU 480 OE1 - LYS 74 NZ ( 14.755 ) | ASP 338 O - ASN 23 ND2 ( 19.687 ) |
| ASP 486 OD2 - TYR 81 OH (18.327) | THR 487 OG1 - TYR 6 N ( 18.627 ) | T2P 451 OE1 - TYR 32 OH ( 23.127 ) |  | GLU 490 OE1 - HIS 35 NE2 ( 20.363 ) | ASP 338 OD1 - LYS 28 NZ ( 16.943 ) |
| PHE 489 N - TYR 81 OH (15.191) | ALA 488 N - TYR 6 OH ( 17.611 ) | ARG 453 NH1 - ILE 45 O ( 21.311 ) |  | THR 491 OG1 - TYR 72 O ( 56.902 ) | ASP 339 N - ASN 23 OD1 ( 13.759 ) |
| PHE 489 N - ASP 83 OD1 (15.159) | ALA 488 N - TYR 72 O ( 18.391 ) | ARG 453 NE - ILE 45 O ( 15.895 ) |  | THR 491 OG1 - GLY 75 N ( 12.835 ) | ASP 339 OD1 - ASN 23 ND2 ( 34.679 ) |
| GLU 490 OE2 - GLN 27 NE2 (15.131) | THR 491 OG1 - TYR 72 O ( 47.422 ) | ARG 453 NE - GLY 48 N ( 26.067 ) |  | SER 492 N - LYS 74 O ( 21.991 ) | ASP 339 OD1 - ASN 23 N ( 28.483 ) |
| GLU 490 N - TYR 81 OH (13.683) | SER 492 OG - HIS 35 O ( 12.787 ) | CYS 485 O - LYS 50 NZ ( 12.3 ) |  | LYS 493 NZ - TYR 6 OH ( 11.156 ) | GLU 342 OE1 - HIS 13 NE2 ( 45.898 ) |
| GLU 490 OE2 - TYR 81 OH (19.103) | SER 492 OG - GLU 37 OE1 ( 19.207 ) | ASP 486 OD2 - LYS 50 NZ ( 25.947 ) |  |  | GLU 342 OE1 - GLN 66 N ( 34.735 ) |
| SER 492 N - TYR 81 OH (18.027) |  | ASP 486 OD1 - LYS 50 NZ ( 10.892 ) |  |  | GLU 342 OE2 - GLN 66 NE2 ( 28.147 ) |
|  |  | ASP 486 OD2 - ARG 52 NE ( 37.734 ) |  |  | GLU 342 OE1 - LYS 85 NZ ( 10.68 ) |
|  |  | THR 487 OG1 - ASP 2 OD1 ( 11.516 ) |  |  | SER 343 N - THR 64 O ( 12.232 ) |
|  |  | ALA 488 N - ASP 2 OD1 ( 34.215 ) |  |  | SER 343 OG - ASP 67 O ( 17.147 ) |
|  |  | PHE 489 N - ASP 2 OD1 ( 32.951 ) |  |  | SER 344 OG - THR 64 O ( 22.531 ) |
|  |  | PHE 489 N - PRO 3 O ( 39.142 ) |  |  | SER 344 O - GLN 72 NE2 ( 10.948 ) |
|  |  | GLU 490 N - ASP 2 OD2 ( 42.71 ) |  |  | ASP 345 OD2 - LYS 71 NZ ( 32.267 ) |
|  |  | GLU 490 N - ASP 2 OD1 ( 12.595 ) |  |  | ASP 345 OD2 - GLN 72 N ( 16.295 ) |
|  |  | THR 491 O - SER 46 OG ( 10.444 ) |  |  | ASP 345 OD1 - GLN 72 NE2 ( 10.516 ) |
|  |  | SER 492 OG - ASP 5 OD1 ( 10.276 ) |  |  | ASP 347 OD2 - SER 16 OG ( 50.854 ) |
|  |  | LYS 493 NZ - ASP 5 O ( 17.375 ) |  |  | ASP 347 OD1 - LYS 85 N ( 32.027 ) |
|  |  |  |  |  | ASN 350 O - LYS 85 N ( 14.123 ) |
|  |  |  |  |  | ASN 350 O - GLU 86 N ( 10.948 ) |
|  |  |  |  |  | SER 351 OG - LYS 85 NZ ( 16.943 ) |
|  |  |  |  |  | LYS 352 N - LYS 85 O ( 29.411 ) |
|  |  |  |  |  | LYS 352 N - GLU 86 OE1 ( 32.371 ) |
|  |  |  |  |  | ASN 353 N - LYS 85 O ( 29.107 ) |
|  |  |  |  |  | ASN 353 N - GLU 86 OE1 ( 27.083 ) |
|  |  |  |  |  | SER 354 OG - THR 20 O ( 21.247 ) |
|  |  |  |  |  | ARG 356 NH2 - ASN 23 ND2 ( 11.072 ) |
|  |  |  |  |  | GLU 375 OE1 - GLN 35 NE2 ( 24.675 ) |
|  |  |  |  |  | GLU 375 OE2 - ARG 49 NH1 ( 19.219 ) |
|  |  |  |  |  | GLU 375 OE1 - LYS 50 N ( 17.119 ) |
|  |  |  |  |  | GLU 375 OE1 - LYS 51 NZ ( 25.363 ) |
|  |  |  |  |  | GLN 376 NE2 - ARG 49 NE ( 36.291 ) |
|  |  |  |  |  | GLU 379 OE1 - LYS 50 N ( 14.851 ) |
|  |  |  |  |  | GLU 379 OE1 - LYS 50 NZ ( 10.54 ) |
|  |  |  |  |  | GLU 379 OE2 - LYS 51 NZ ( 45.242 ) |
|  |  |  |  |  | GLU 379 OE1 - LYS 51 N ( 42.67 ) |
|  |  |  |  |  | GLU 379 OE2 - LYS 51 N ( 11.536 ) |
|  |  |  |  |  | GLU 379 OE1 - ARG 52 N ( 38.19 ) |
|  |  |  |  |  | GLU 379 OE2 - ARG 52 NH1 ( 19.303 ) |
|  |  |  |  |  | ARG 382 N - ARG 52 NH1 ( 17.139 ) |
|  |  |  |  |  | SER 418 OG - ARG 49 NH2 ( 11.224 ) |
|  |  |  |  |  | ARG 453 NH1 - SER 46 OG ( 17.291 ) |
|  |  |  |  |  | ASP 486 OD2 - LYS 50 NZ ( 18.559 ) |
|  |  |  |  |  | ASP 486 OD2 - ARG 52 NE ( 41.782 ) |
|  |  |  |  |  | ALA 488 N - ASP 5 OD1 ( 53.442 ) |
|  |  |  |  |  | ALA 488 O - TYR 47 OH ( 10.66 ) |
|  |  |  |  |  | PHE 489 N - ASP 5 OD2 ( 46.626 ) |
|  |  |  |  |  | THR 491 OG1 - TYR 47 OH ( 15.571 ) |
