# Supplementary material for: Viral Evolved Inhibition Mechanism of the RNA Dependent Protein Kinase PKR's Kinase Domain, a Structural Perspective
Source: PLoS One. 2016 Apr 18;11(4):e0153680. doi: 10.1371/journal.pone.0153680 (PMC4835081; doi:10.1371/journal.pone.0153680)
Supplement: S3 Table — The percentages of PKR residues and the Aloop residues in different secondary structural regions in the six protein complexes. A major change can be observed in the 310 helix formations of the Aloop. (DOCX) [file pone.0153680.s004.docx]

**S3 Table. Secondary structural variations of PKR protein.** The percentages of PKR residues and the Aloop residues in different secondary structural regions in the six protein complexes. A major change can be observed in the 3_10_ helix formations of the Aloop.

|  | **Coil** | **β-Sheet** | **β -Bridge** | **Bend** | **Turn** | **α-Helix** | **5-Helix** | **3_10_-Helix** |
| --- | --- | --- | --- | --- | --- | --- | --- | --- |
| **Structural variations of PKR protein** |  |  |  |  |  |  |  |  |
| PKR_pp_-eIF2α | 22 | 18 | 1 | 13 | 13 | 32 | 0 | 1 |
| PKR_pp_-K3L | 21 | 19 | 1 | 12 | 11 | 35 | 0 | 1 |
| PKR_pp_-TAT | 22 | 18 | 1 | 10 | 11 | 36 | 0 | 1 |
| PKR_p_-eIF2α | 23 | 17 | 1 | 12 | 12 | 34 | 0 | 1 |
| PKR_p_-K3L | 21 | 19 | 1 | 10 | 12 | 35 | 0 | 1 |
| PKR_p_-TAT | 21 | 20 | 1 | 12 | 10 | 35 | 0 | 1 |
| **Aloop structural variations** |  |  |  |  |  |  |  |  |
| PKR_pp_-eIF2α | 62 | 0 | 0 | 23 | 10 | 0 | 0 | 5 |
| PKR_pp_-K3L | 67 | 0 | 0 | 28 | 5 | 0 | 0 | 0 |
| PKR_pp_-TAT | 61 | 0 | 0 | 22 | 12 | 0 | 0 | 6 |
| PKR_p_-eIF2α | 63 | 0 | 0 | 23 | 13 | 0 | 0 | 0 |
| PKR_p_-K3L | 65 | 0 | 0 | 25 | 9 | 0 | 0 | 1 |
| PKR_p_-TAT | 61 | 0 | 0 | 25 | 13 | 0 | 0 | 1 |
