# Supplementary material for: Viral Evolved Inhibition Mechanism of the RNA Dependent Protein Kinase PKR's Kinase Domain, a Structural Perspective
Source: PLoS One. 2016 Apr 18;11(4):e0153680. doi: 10.1371/journal.pone.0153680 (PMC4835081; doi:10.1371/journal.pone.0153680)
Supplement: S4 Table — Residues involved in the domain formation, which helps the PKR protein to rotate, and their effective rotational angles are indicated. (DOCX) [file pone.0153680.s005.docx]

**S4 Table. Domain motion contributing residues.** Residues involved in the domain formation, which helps the PKR protein to rotate, and their effective rotational angles are indicated.

| **Protein Complex** | **Domain no. &**  **Rotational angle in degrees** | **Residues involved** |
| --- | --- | --- |
| **PKR_pp_-eIF2α** | Domain 1  (12.00°) | 266, 267, 268, 269, 270, 271, 272, 273, 280, 281, 282, 283, 284, 285, 286, 287, 288, 289, 291, 292, 293, 294, 295, 296, 297, 298, 299, 300, 301, 302, 303, 304, 305, 306, 307, 308, 309, 310, 312, 323, 324, 325, 330, 361, 362,, 363, 364, 365, 366, 367, 412, 414, 415, 416, 417, 418, 419, 420, 429, 430, 431, 432, 433, 434, 435, 436 |
|  | Domain 2  (20.96°) | 326, 327, 328, 329, 331, 332, 333, 334, 335, 336, 337, 339, 340, 341, 342, 354, 355, 356, 357, 358, 359, 360, 413, 451 |
| **PKR_pp_-K3L** | Domain 1  (12.27°) | 262, 270, 271, 272, 273, 280, 281, 282, 283, 330, 331, 332, 333, 334, 335, 336, 337, 338, 339, 340, 341, 342, 355, 356, 357, 358, 359, 360, 367, 369, 370 |
| **PKR_pp_-TAT** | Domain 1  (19.04°) | 267, 268, 269, 270, 271, 272, 273, 274, 279, 280, 281, 282, 283, 284, 285, 286, 291, 292, 293, 294, 295, 296, 297, 298, 299, 300, 323, 324, 325, 326, 327, 329, 330, 331, 332, 333, 334, 335, 336, 337, 356, 357, 358, 359, 360, 361, 362, 363, 364, 365, 366, 368 |
| **PKR_p_-eIF2α** | Domain 1  (16.99°) | 263, 264, 265, 266, 267, 268, 269, 270, 271, 272, 273, 278, 279, 280, 281, 282, 283, 284, 285, 286, 287, 292, 293, 294, 295, 296, 297, 298, 299, 300, 320, 321, 322, 323, 324, 326, 327, 328, 329, 330, 331, 332, 333, 334, 335, 357, 359, 360, 361, 362, 363, 364, 365, 366, 367, 368, 369, 370, 372, 373, 374, 417, 419, 420, 421, 432, 471, 472, 473 |
|  | Domain 2  (11.74°) | 275, 276, 288, 289, 290, 291, 301, 302, 303, 304, 305, 315, 317, 336, 408, 409, 410, 434, 438, 439, 440 |
| **PKR_p_-K3L** | Domain 1  (11.94°) | 303, 316, 317, 369, 370, 371, 372, 399, 402, 403, 404, 405, 406, 407, 408, 409, 410, 411, 413, 421, 423, 429, 431, 432, 433, 447, 448, 449, 450, 451, 452, 453, 454, 455, 456, 457, 461, 462, 463, 469, 470, 471, 472, 473, 474, 475, 516, 518, 519, 520, 525, 526, 527, 528, 529, 530, 531, 532, 533, 534, 535, 536, 537 |
|  | Domain 2  (6.04°) | 263, 264, 266, 267, 268, 269, 270, 271, 272, 273, 274, 275, 277, 278, 282, 287, 342, 343 |
| **PKR_p_-TAT** | Domain 1  (10.39°) | 303, 304, 305, 306, 307, 308, 309, 310, 311, 312, 313, 314, 315, 316, 317, 320, 321, 350, 351, 352, 383, 384, 386, 387, 388, 389, 390, 391, 392, 393, 394, 395, 396, 401, 403, 404, 405, 406, 407, 408, 414, 418, 419, 422, 429, 430, 431, 432, 433, 434, 445, 454, 456, 467, 468, 469, 470, 471, 472, 473, 474, 475, 476, 495, 497, 498, 503, 507, 508, 509, 510, 511, 512, 513, 514, 515, 516, 517, 518, 519, 520, 521, 526, 527, 528, 529, 530, 531, 532, 533, 534, 535, 536, 537, 538, 539, 540 |
|  | Domain 2  (13.16°) | 263, 284, 285, 286, 288, 289, 290, 291, 292, 293, 339, 341, 342, 343, 346, 347, 348, 367, 368, 369, 423, 424, 425 |
